# Electronic health record modification and dashboard development to improve clinical care in pediatric rheumatology

**DOI:** 10.3389/fped.2024.1428792

**Published:** 2024-08-13

**Authors:** Livie Timmerman, Heather Dutton, Nicholas McDannald, Emily A. Smitherman, Melissa L. Mannion

**Affiliations:** ^1^ Department of Pediatrics, University of Alabama at Birmingham, Birmingham, AL, United States; ^2^ Information Technology, Children’s of Alabama, Birmingham, AL, United States

**Keywords:** electronic health record, dashboard, population management, juvenile idiopathic arthritis, pediatric rheumatology

## Abstract

**Objective:**

This report describes our experience in electronic health record (EHR) note modification and creation of an external dashboard to create a local learning health system that contributes to quality improvement and patient care within our pediatric rheumatology clinic.

**Methods:**

We applied quality improvement methodology to develop a more reliable and accurate system to identify patients with juvenile idiopathic arthritis and track important measures that aide in improving patient care and performance outcomes. From 2019 to 2021, we iteratively modified our outpatient clinic EHR note to include structured data elements to improve longitudinal monitoring. We then validated data transferred to an electronic dashboard external to the EHR and demonstrated utility for identifying an accurate patient population and tracking quality improvement initiatives.

**Results:**

Creation of the structured data elements improved the identification of patients with JIA with >99% accuracy and without requiring manual review of the chart. Using the dashboard to monitor performance, we improved documentation of critical disease activity measures that resulted in improvement in those scores across the local population of patients with JIA. The structured data elements also enabled us to automate electronic data transfer to a multicenter learning network registry.

**Conclusion:**

The structured data element modifications made to our outpatient EHR note populate a local dashboard that allows real time access to critical information for patient care, population management, and improvement in quality metrics. The collection and monitoring of structured data can be scaled to other quality improvement initiatives in our clinic and shared with other centers.

## Introduction

Technology advancement and universal use of electronic health records (EHR) has allowed providers new ways to collect and track quality measures and improvements within healthcare. While the purpose of the health record is to document medical care, an electronic record can be leveraged to capture specific aspects of care and serve as a tool to efficiently access and analyze care processes, specific disease measures, and health outcomes (1, 2). These quality measures serve as benchmarks for evaluating the effectiveness, safety, and efficiency of health care services, facilitating the monitoring and improvement of clinical practices and patient outcomes (3).

Routine measurement and monitoring of clinical disease activity and care processes is especially important for patients with chronic diseases and is facilitated by an easy-to-use system (4). The most common type of chronic arthritis among children is juvenile idiopathic arthritis (JIA), an autoimmune disease affecting approximately 1 in 1,000 children (5). Individuals with JIA require longitudinal treatment to reduce complications of inflammation and have frequent healthcare interactions for evaluation of disease activity, medication toxicity monitoring, and screening for extraarticular manifestations like asymptomatic uveitis (6–9). Pediatric rheumatologists use various clinical measures to assess disease activity, treatment efficacy, and quality of life (10). These measures have been utilized in studies of a patient-facing dashboard to facilitate patient education and shared decision-making in pediatric rheumatology studies (11, 12).

Clinical outcomes and quality measure performance can be monitored in a clinic population or across clinical sites within the infrastructure of a learning network (13). For example, the Pediatric Rheumatology Care and Outcomes Improvement Network (PR-COIN) is a pediatric rheumatology specific quality improvement learning network that has a centralized patient registry with a dashboard to display network and site-specific processes and disease activity outcomes for patients with JIA (14, 15). Participating sites contribute patient-specific data to the centralized registry with the goal of capturing every JIA patient at every visit to allow for population management (14), a process that has been facilitated by the use of electronic data capture from the EHR with automatic uploading to the registry.

The goal of this initiative was to create an efficient and accurate process for identifying patients with JIA in our EHR, access and track key metrics relate to patient outcomes and clinical care decisions for patients with JIA and automate structured data transfer to the PR-COIN Registry. With the advisement and collaboration of our Information Technology (IT) department, we were able to modify our EHR documentation, create an external dashboard using EHR data that updates in real time, and utilize electronic data transfer to contribute data from our local population to a centralized multicenter registry. We detail our experience with iterative note modifications to create structured data elements within the EHR, utilizing a clinic dashboard for monitoring quality metrics in our population of patients with JIA, and automating data transfer to a multicenter learning network registry.

## Methods

### Context

**Figure 1:**
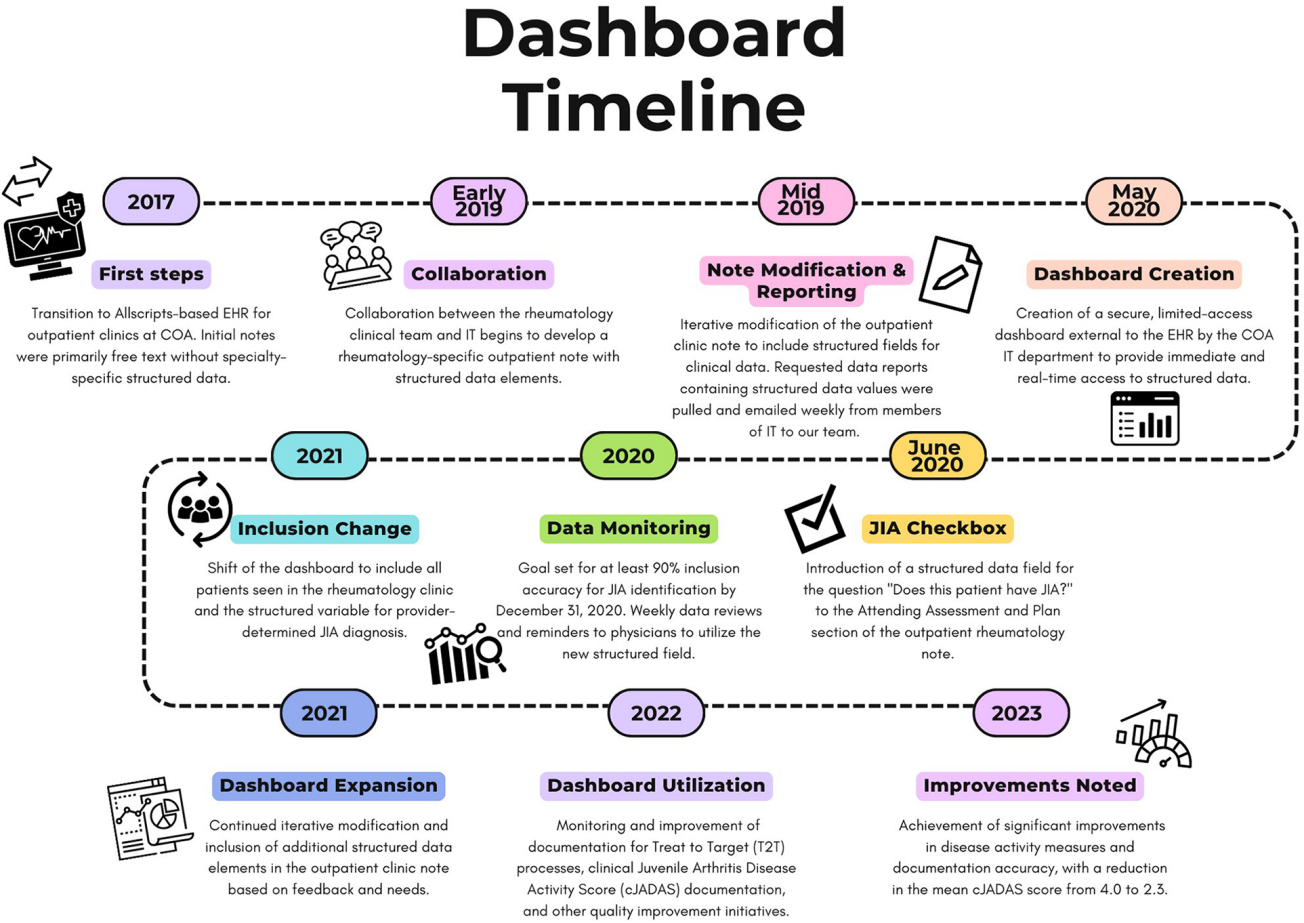
Timeline of initiative to improve documentation and review of clinical metrics for JIA in the UAB/COA pediatric rheumatology clinic.

Children's of Alabama (COA) is a tertiary care children's hospital in conjunction with the University of Alabama at Birmingham (UAB) that provides comprehensive specialty and subspecialty care for the children of Alabama. Our COA/UAB pediatric rheumatology clinic serves approximately 225 patients each month with varying rheumatic conditions. The outpatient clinics at COA were transitioned to an Allscripts-based EHR in September 2017, and specialty-specific note customization began in 2019 (Figure 1). Our clinical care team at the time of this initiative consisted of 5 attending physicians, 2 clinical pediatric rheumatology fellows, 4 nurse practitioners, and 5 registered nurses. Our clinic is also supported by 4 administrative staff, a medical social worker, and a dedicated research coordinator. Prior to this initiative, there was not a standardized method of identifying patients with JIA so identification of patients for research and abstraction of disease activity measures were performed manually by the providers or research coordinator.

### EHR modification and patient identification

After transition to the new EHR system, we received weekly reports from IT that identified patients with JIA by International Classification of Diseases- 10th edition (ICD-10) codes. However, these data requests then required manual review for completeness and accuracy. At the time of the outpatient EHR launch in September 2017, outpatient clinic notes were primarily free text without specialty specific structured data. The rheumatology clinic utilized a specialty-specific note that was developed for inpatient use that included a few clinical data and specific quality measures available in structured fields. For the patients with JIA identified by IT using ICD-10 codes, these data elements (active joint count, physician global assessment of disease activity, patient/parent global assessment of well-being) were provided in the weekly reports emailed by a member of the IT team. However, the measures that were included in the report were often incomplete which limited our ability to monitor and improve care processes and outcomes. Another challenge to analyzing these clinical reports, was that the patients identified by IT with ICD-10 codes resulted in both false positive and false negative results and did not accurately reflect our JIA population. In our clinic the diagnosis codes for billing were collected on paper and the problem lists in the EHR were not required to complete documentation. For each report, every included patient required chart review to confirm a primary diagnosis of JIA and the clinic list for each week would be manually reviewed for possible missed JIA patients.

Beginning in 2019, the rheumatology clinical team and IT collaborated to develop a rheumatology-specific outpatient note that contained structured data elements that were important to clinical care, quality improvement initiatives, and research-based registries (Figure 1). Over the span of two years, we iteratively modified our rheumatology-specific outpatient clinic note to include structured fields (Table 1) for clinical data that would allow for longitudinal monitoring of patients with JIA for quality improvement initiatives and to facilitate data collection for research. We identified key metrics as outlined by the American College of Rheumatology guidelines for treatment of JIA (6, 9, 16, 17), quality metrics identified by PR-COIN (14, 15), and measures important to our clinical team to build data fields needed to capture these measures from the EHR (Table 1). These measures are collected as part of routine clinical care to determine disease activity and make treatment decisions and include the components and calculated clinical juvenile arthritis disease activity score (cJADAS), calculation of recommended eye screening, and attestation of the components of treat to target (T2 T). The cJADAS is a composite score that includes the physician global assessment of disease activity, the parent/patient assessment of overall well-being, and a count of active joints (maximum of 10). This score ranges from 0 to 30, with higher scores indicating greater disease activity. The goal of T2T is to utilize regular measurement of a standardized assessment and use shared decision making with families to make treatment changes in order to achieve and maintain the lowest possible disease activity to prevent long-term joint damage and improve quality of life (18).

**Table 1:** Table of all structured data collected in the UAB/COA pediatric rheumatology clinical outpatient note and dashboard.

Structured data measure	Structure	Available in clinical note	Available on dashboard
All Visits			
Telehealth	Radio	Y	N
Morning stiffness (none, </= 15 min, >15 min)	Radio	Y	N
Inflammatory back pain (Y/N)	Radio	Y	N
Date of last eye exam	Date	Y	N
Last eye exam results (active, past, no uveitis to date)	Radio	Y	N
TB testing date	Date	Y	Y
Hepatitis testing date	Date	Y	N
Home medication	Text	Y	Y
Sum of weight(kg)	Numeric	Y	Y
BMI	Calculated	Y	Y
BP	Numeric	Y	Y
Mouth opening	Numeric	Y	N
Jaw deviation with opening	Radio	Y	N
Notable micrognathia	Radio	Y	N
Modified schober's	Numeric	Y	N
flat back	Radio	Y	N
Scoliosis	Radio	Y	N
Gait (normal/abnormal)	Radio	Y	N
leg length discrepancy (Y/N)	Radio	Y	N
Enthesitis (list for right and left separately: superior patella, inferior patella, Achilles insertion, plantar fascia insertion, metatarsal heads, tibial tuberosity, greater trochanter of femur, elbow condyles)	Radio	Y	N
Left enthesitis count	Calculated	Y	N
Right enthesitis count	Calculated	Y	N
joint assessment (left and right active joint for 72 joints)	Radio	Y	N
Joint assessment (left and right decreased ROM for 72 joints)	Radio	Y	N
Active joint count	Calculated	Y	Y
Decreased ROM count	Calculated	Y	N
transition discussed (Y/N)	Radio	Y	Y
TRAQ score	Numeric	Y	Y
Current glucocorticoid use (Y/N)	Radio	Y	Y
Glucocorticoid type (Oral, IV, other)	Radio	Y	Y
Disease activity assessment (inactive, mild, moderate, severe)	Radio	Y	Y
Pain scale (0–10)	Numeric	Y	N
CHAQ score	Numeric	Y	Y
MD global	Numeric	Y	Y
Parent global	Numeric	Y	Y
cJADAS10 value	Calculated	Y	Y
Treatment target set with family at this visit (Y/N)	Radio	Y	Y
Date target set	Date	Y	N
Target assessment (at target, not at target)	Radio	Y	Y
Disease management change at this visit (Y/N)	Radio	Y	Y
Shared decision-making aid used	Radio	Y	N
Self-management support provided (Y/N)	Radio	Y	N
JIA			
Has JIA ChronicDx (ICD-10 based)	Calculated	N	Y
JIA Yes/No	Radio	Y	Y
JIA subtype (systemic, persistent oligoarticular, extended oligoarticular, oligoarticular unspecified, RF + polyarticular, RF- polyarticular, ERA, psoriatic, undifferentiated)	Drop Down	Y	Y
ANA (+/-)	Radio	Y	N
RF (+/-)	Radio	Y	N
CCP (+/-)	Radio	Y	N
HLA B27 (+/-)	Radio	Y	N
Prognostic features (hip arthritis, wrist arthritis, ankle arthritis, C-spine involvement, radiographic damage, sacroiliitis, TMJ arthritis)	Radio	Y	N
Date of JIA diagnosis	Date	Y	Y
Age at diagnosis (Years)	Calculated	Y	N
Duration of diagnosis (Years)	Calculated	Y	N
Recommended eye screening interval	Calculated	Y	N
JIA symptoms (present/absent)	Radio	Y	N
Systemic JIA symptoms present (fever, rash, serositis, splenomegaly, generalized lymphadenopathy)	Radio	Y	N
Inflammatory markers (elevated, normal, unknown)	Radio	Y	N
Active uveitis (present, absent, unknown)	Radio	Y	N
Morning stiffness >15 min (Y/N)	Radio	Y	N

TB, *Mycobacterium tuberculosis*; kg, kilograms; BMI, body mass index; BP, blood pressure; ROM, range of motion; TRAQ, transition readiness assessment questionnaire; IV, intravenous; CHAQ, childhood health assessment questionnaire; MD global, physician global disease activity assessment; parent global—parent/patient assessment of well-being; cJADAS10, clinical juvenile arthritis disease activity score 10 joint count; JIA, juvenile idiopathic arthritis; ICD-10, International Classification of Diseases 10th edition; RF, rheumatoid factor; ERA, enthesitis related arthritis.

**Figure 2:**
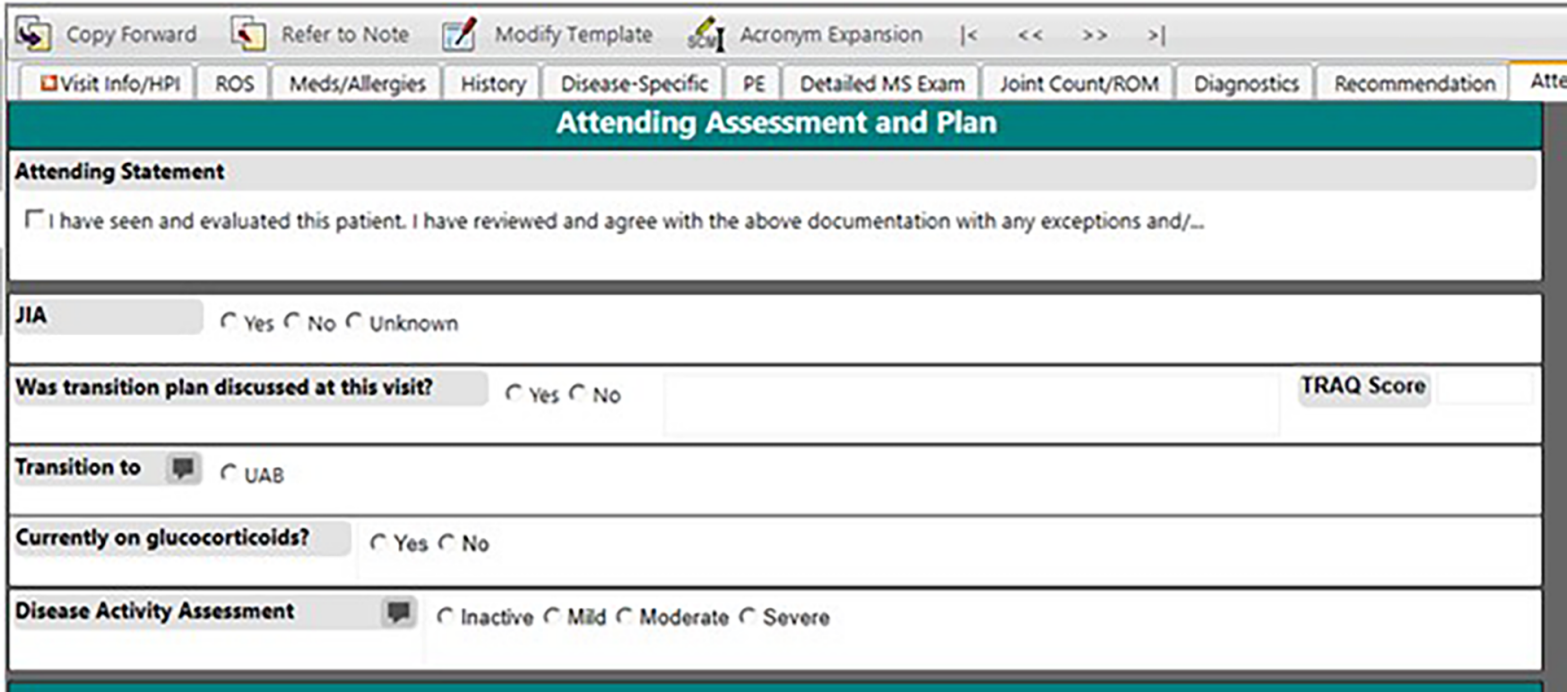
Screenshot of the EHR clinical note template with the provider-determined JIA field.

Since relying on ICD-10 code-based definitions led to an inaccurate JIA population, we decided to create a structured field within our note for providers to attest JIA diagnosis. This eliminated the need to manually review query results. In June 2020, we added a structured data field for the question “Does this patient have JIA?” to the Attending Assessment and Plan section of the outpatient rheumatology note (Figure 2). We introduced the new identification process to our providers and set a goal of at least 90% inclusion accuracy by December 31, 2020, giving us approximately 6 months to adjust. Eligible patients included both new and return rheumatology patients with all of the JIA subtypes including oligoarticular, polyarticular (rheumatoid factor positive and negative), enthesitis-related, psoriatic, undifferentiated, and systemic JIA (19). At the visit patients were classified into three categories: JIA yes, JIA no, or unknown. Each selection carried forward to the next clinic note so that the provider only had to mark the appropriate classification once and update if necessary (i.e., if a patient was marked unknown as a new patient and was later determined to have JIA, then the provider would update classification to “JIA yes” at the next visit). Our team utilized various improvement science techniques including statistical run charts and Plan-Do-Study-Act (PDSA) cycles to test changes and track accuracy of the JIA specific checkbox documentation (20). To monitor our progress, we reviewed the data weekly, comparing the patient population identified by ICD-10 codes and the “JIA checkbox” to manual review of the EHR for all patients seen in clinic each week to evaluate the accuracy of patient identification. We tracked the patients in which “JIA yes” should have been checked and sent out weekly reminders to the providers and clinical staff to complete at that patient's next visit. We also put reminder notes on all the computers in the clinic workroom to utilize the new structured field. Once we consistently had more than 90% accuracy for the “JIA checkbox”, we abandoned comparing this list to the patient list based on ICD-10 codes.

### Dashboard development

**Figure 3:**
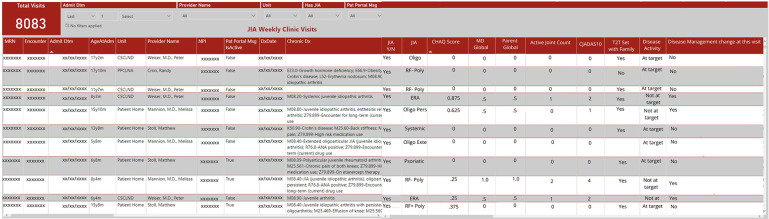
Screenshot of local dashboard populated by EHR data.

As IT's resources became overburdened, our weekly rheumatology query result delivery became inconsistent and the EHR did not allow for clinical personnel to perform data queries. Given our limited IT resources and the time constraints for manually extracting data, combined with the need for frequent and ongoing data updates to continuously improve patient care and quality improvement initiatives, we identified an alternative approach (Figure 1). In May 2020, the COA IT department created a secure, limited-access dashboard external to the EHR to provide immediate and real time access to the structured data that had previously been shared by weekly emailed data query reports (Figure 3). The dashboard was designed based on input from providers to prioritize critical data points such as cJADAS10 scores and medication usage. It provided the flexibility to add, drop, and modify metrics as needed based on our current monitoring initiatives and the most current literature and recommendations. Not all structured data elements were included on the dashboard as they all did not require population monitoring on a frequent basis. The column order of variables were determined by IT based upon the data extraction. However, the dashboard structure allowed us to export data to other programs in spreadsheet or comma separated variable format. This function allowed us to better sort, visualize, and share data using graphs and tables.

Initially the dashboard was populated by patients with JIA identified using ICD-10 codes, with similar results to the weekly data reports. With an expanding portfolio of quality improvement projects, we shifted the dashboard to include all patients seen in the rheumatology clinic and included the structured variable for provider determined JIA diagnosis. This adjustment allowed us to filter on “JIA yes” for continued monitoring of accuracy and completeness of patient identification and JIA-specific metrics. Inclusion of all patients seen in rheumatology clinic also allowed us to monitor other structured data from the clinic note which iteratively led to the modification and inclusion of additional structured data elements in the note.

## Results

By modifying our outpatient rheumatology note in the EHR to include structured data elements and a JIA diagnosis attestation specific field, we were able to accurately identify our JIA patient population and collect disease activity measures while abandoning the use of manual chart abstraction. We identified an average of 309 patients per quarter, all subtypes were represented, and ages ranged from 12 months to 20 years. Encounters from our main clinic site as well as offsite and telemedicine visits were all included. Within 3 months of creation of the “JIA checkbox”, we achieved and maintained 99% accuracy for identifying patients with JIA. These structured data elements also facilitated the development of an external dashboard based on the EHR that updates in real time to allow us to monitor documentation, track quality improvement initiatives, and eliminate the need for recurring data requests. By easily monitoring quality and disease measures in a specific population, we can track outcomes and processes that have been implemented into our clinic (Figure 3). The structured data elements are also automatically extracted to populate an external learning network registry to further our local and national improvement initiatives. COA/UAB pediatric rheumatology was the first and only site with a non-Epic EHR to successfully implement electronic data transfer to the PR-COIN Registry.

In addition to improving and maintaining reliable utilization of the “JIA checkbox” to identify an accurate JIA population, our team used the structured data elements and available dashboard for other improvement initiatives. For example, in conjunction with PR-COIN (21), we began to monitor use of treat to target (T2 T) processes for our patients with JIA. This included setting a treatment goal with the patient and family, calculating the cJADAS at the point of care, and assessing whether the patient was at goal or not at goal. During the course of the network-wide initiative, we were able to increase documentation of a T2T goal from 0 to 85% of visits for JIA and improved cJADAS documentation from 17 to 88% of visits. We accomplished this through weekly monitoring, multiple PDSA cycles, and division-wide celebrations for achieving our smart aim goals. Following this initiative, by improving our measurement of cJADAS and documenting a T2T goal, we had an overall reduction in the mean cJADAS across our JIA population, from 4.0 in April 2019 to 2.3 in January 2023. This reduction indicates an overall improvement in disease activity including patient outcomes, reflecting the effectiveness of measuring a disease activity score in achieving lower disease activity levels that is seen and recommended in other diseases like rheumatoid arthritis (22).

## Discussion

The structured collection of data, frequent monitoring, and continuous improvement of quality metrics are crucial elements in the modern healthcare setting, especially in managing chronic conditions like JIA. This initiative demonstrates how advancements in EHR systems can significantly enhance the management and treatment outcomes of such diseases through efficient data utilization. We customized our rheumatology outpatient EHR note with structured data fields to populate a real-time dashboard that enabled us to improve documentation of quality metrics and improve disease activity measures in our JIA patient population. By utilizing the EHR to collect electronic clinical quality measures that have been tailored to our practice, automatic extraction allowed for more efficient generation of performance measures. The dashboard allows for frequent performance updates and development of targeted improvement strategies.

We demonstrated improvement in documentation of the components of the cJADAS and T2T goal setting for patients with JIA and saw an improvement in average cJADAS across our JIA population over time. This reduction indicates that patients are experiencing fewer symptoms and less severe disease activity, overall contributing to better disease control and improved quality of life for the population. While difficult to interpret the significance of change for an individual in this population since there are patients with both oligo- and polyarticular disease, the reduction in score for the population is an important metric for quality care in the clinic (14). Improvement in the population cJADAS could be a result of improved disease activity or improved health related quality of life (23) through optimized treatment, increased response to adverse effects of medication, addition of non-pharmacologic treatments, increased education of the score itself, and awareness of monitoring by both patients and providers resulting in a social desirability bias. Frequent disease activity assessment is a component of the recommended T2T approach for the management of JIA to quickly achieve disease control and limit long-term complications of disease (18). Other components include target disease activity setting with the patient, and treatment changes to achieve the disease activity target (18). Disease activity measures can be used for each individual at the point of care in a T2T approach (21), but these measures can also be used to assess the disease activity of a clinic population in evaluation of overall quality of care. Importantly, by monitoring our documentation performance we were able to maintain high levels of T2T goal setting even after the primary intervention ended.

The introduction of a JIA-specific attestation field within the outpatient rheumatology notes has not only streamlined the process of patient identification but has also reduced the inaccuracies associated with the reliance on ICD-10 codes alone. Initial efforts to collect and monitor metrics were time consuming because of limited resources and resulted in a lack of consistent and accurate data. Because ICD-10 codes and administrative claims are not always the most accurate way of determining primary disease (24), we improved our process for identifying patients with JIA within our local EHR. Implementation of modified EHR systems has been shown to streamline the documentation process, standardize data entry procedures, and improve data accuracy and completeness. These modifications have been instrumental in addressing longstanding challenges associated with manual documentation, such as illegibility, inconsistency, and fragmentation of patient records (1, 4, 25). The addition of other structured data fields allowed for critical information such as disease activity, treatment responses, and patient well-being to be consistently recorded and easily accessible. This structured approach facilitates more accurate population health management and individual patient care, highlighting its significance in clinical settings (1, 26).

Dashboards are a tool used to extract data to make information easily accessible to the user. They originally were primarily utilized in the marketing field; however recently they have been modified to become a valuable resource in the healthcare field (4). Dashboards can present individual or population level information in a timely manner and can be flexible to allow inclusion of metrics that are important to the department, clinic, and patients. The information displayed can demonstrate change in one measure over time, change in measures in response to an intervention, or a cross sectional assessment of several measures at one time (4, 27–30). In this case we utilized the population-based real-time dashboard to identify interventions for rapid Plan-Do-Study-Act cycles and monitored documentation performance and subsequent changes in clinical outcomes for the cohort.

Moreover, the structured nature of EHR templates allows for standardized data capture, facilitating easier aggregation and analysis of clinical data for research and quality improvement purposes. As a result, healthcare organizations have been able to leverage EHR-derived data to monitor clinical performance, identify areas for improvement, and implement targeted interventions to enhance the quality and safety of patient care delivery (4, 29). Through the systematic collection of patient data, we were able to generate comprehensive datasets to monitor our documentation performance and evaluate patient outcomes over time to identify areas for improvement to enhance the quality and safety of patient care delivery. Data collected during routine clinical care not only benefits individual patients but also contributes to broader research and quality improvement efforts aimed at enhancing healthcare outcomes on a population level. We also included structured data fields critical for the PR-COIN Registry, initially for ease of manual data entry, but the structured format allowed for automated electronic data transfer.

Challenges to the development and continued utilization of the electronic dashboard include resources to be able to make necessary changes, continuation of processes, and limited data availability within the EHR. With the lack of resources available to address concerns or problems as they arise, we may wait weeks for problems to be resolved. Other limitations that exist include dependency of providers to continue to mark the disease specific attestation checkbox for patients. Although we achieved >90% accuracy and providers only need to mark yes once, new diagnoses will require providers to continue to participate in this process. We have continued to monitor accuracy monthly and remind providers as needed to sustain our performance. Additionally, our data collection is limited to the information that has been collected in the current EHR, introduced to our local outpatient clinics in 2017. Any data from prior to 2017 still requires manual chart abstraction and is subject to high rates of missing data.

The advancements in EHR technology and its application in our local pediatric rheumatology clinic exemplify the potential of digital health solutions to revolutionize medical care. Structured data collection ensures that relevant clinical information is accurately captured and organized, supporting comprehensive and personalized patient care. Frequent monitoring through innovative tools like dashboards enables real-time data analysis, essential for effective disease management and intervention adjustments. The focus on quality metric improvement helps in refining clinical practices and enhancing patient outcomes. We have continued to modify the outpatient note to create structured values that will transfer into the dashboard as needed to meet the needs of various QI projects and research studies. The ability to measure and evaluate performance in real-time will allow us to improve other quality metrics in other patient populations.

## Data Availability

The datasets presented in this article are not readily available because these data are directly linked to the EHR and are not deidentified outside of the counts presented in the article. Requests to access the datasets should be directed to Melissa Mannion, mmannion@uabmc.edu.
